# Chewing loads alter TMJ metabolism and Zn homeostasis via PIEZO1/ TRPV4

**DOI:** 10.1186/s13036-025-00564-2

**Published:** 2025-11-28

**Authors:** Yongmei Wang, Brandon H. Lee, Zhiyuan Yang, Haochen Ci, Bo Wang, Joshua Levy, Sunita P. Ho

**Affiliations:** 1 Department of Preventive and Restorative Dental Sciences, School of Dentistry, University of California, 513 Parnassus Avenue, HSW 813, San Francisco, CA 94143 USA; 2 State Key Laboratory of Structural Analysis for Industrial Equipment, Department of Engineering Mechanics, International Research Center for Computational Mechanics, Dalian University of Technology, Dalian, 116023 China; 3 Department of Pathology and Computational Biomedicine, Cedars Sinai, Los Angeles, CA USA; 4 Department of Urology, School of Medicine, University of California, San Francisco, CA 94143 USA

**Keywords:** Chewing loads, Zinc, PIEZO1, TRPV4, Mitochondria, ROS

## Abstract

The biomechanical effects of bite forces on a temporomandibular joint within the craniofacial masticatory complex remain unclear. Herein, we will discuss the effect of recovering a hypofunctioning temporomandibular joint using normal bite forces. Specifically, we will correlate spatiotemporal: **(1)** biomolecular expressions to **(2)** physical characteristics of the inner and outer regions of the temporomandibular condyles. In this study, we divided twelve male Sprague-Dawley rats into three groups: hard-food (*N* = 3, hard pellets), soft-food (*N* = 3, soft-chow – hard-pellets grounded to powder), and recovery (*N* = 6, rats on soft-food for 9 weeks and switched abruptly to hard-food for an additional 8 weeks). Rats were euthanized at 17 weeks, and their condyles were imaged using a preclinical micro-computed tomography to estimate bone mineral density and volume fraction. The condyles were prepared for histology (proteoglycans using Safranin O counterstained with Fast Green, and matrix using Picrosirius Red). We performed immunolocalization of mechanosensory ion channels, PIEZO1 and TRPV4, hypoxia inducible factor-1α (HIF-1α), Indian hedgehog (Ihh), mitofusin 2 (MFN2), and cell senescence (p16). Analogous to in vivo conditions, the PIEZO1 and TRPV4 MS-ion channels of the ATDC5 cells were mechanically activated at 5 and 7 days in vitro. Corresponding agonists and antagonists were used for activation of the mechanosensory ion channels. Staining for zinc (Zn) alongside reverse transcription quantitative polymerase chain reaction (RT-qPCR) for quantification of PIEZO1 and TRPV4, superoxide dismutase 2 (SOD2), dynamin-related protein -1 (DRP-1), metal transcription factor-1 (MTF-1), HIF-1⍺, and metabolism (MFN2), Zn transporters1, 5, and 8 (ZnT1, ZnT5, ZIP8) were performed. The oxygen sensor HIF-1α was associated with regional metabolism, as indicated by MFN2, SOD2, and DRP expressions in proliferating (Ihh) and senescent (p16) cells across all groups. Increased Zn levels, along with MTF-1 and ZIP8 and ZnT1/5, were correlated with PIEZO1 in the proliferative zone, and TRPV4 in the inner hypertrophic and subchondral zones of the condyle. These temporal biochemical changes correlated with increased widths of the hypertrophic region and cartilage with a matrix structure loss but recovered bone mineral density and bone volume fraction in condyles of the recovery group. Results illustrate biochemical changes in the temporomandibular condyle in response to a change in bite force. A major finding with significant novelty is that the mechanically induced biochemical changes involve micronutrient Zn metabolism in relation to the activation of two mechanosensory ion channel proteins PIEZO1 and TRPV4. Hard food stimulation of cells that also are HIF-1α positive can alter Zn levels regulated by Zn transporters; a proposed mechanochemo cascade necessary for HIF-1α regulation and subsequently condylar function. In summary, though the mechanical properties are not yet known, chewing hard foods may help regain cartilage and bone thickness lost through TMJ disuse or chewing on soft chow only.

**Supplementary Information:**

The online version contains supplementary material available at 10.1186/s13036-025-00564-2.

## Introduction

The dental and craniofacial masticatory system consists of multiple joints, including the dentoalveolar fibrous joint (DAJ) and the temporomandibular diarthrodial joint (TMJ). In modern society, processed (soft) diets lead to hypofunction of both the DAJ and TMJ [20]. Conversely, hyperfunction, often seen in bruxism, is also thought to alter TMJ biomechanics and increase the risk of temporomandibular joint osteoarthritis (TMJOA) [3, 24]. Specifically, reduced mastication in nonhuman animals has been linked to thinner condylar cartilage and decreased subchondral bone, both major hallmarks of TMJOA [27]. These observations suggest that normal loading is essential for maintaining the mechanical behavior and homeostasis of condylar cartilage, subchondral bone, and trabecular bone to prevent the onset of TMJOA.

Both under- and over-loading of the teeth trigger rapid mechanotransduction via tissue-specific mechanosensory ion (MS-ion) channels [1, 4] within the masticatory complex. Two MS-ion channels, PIEZO1 and transient receptor potential vanilloid4 (TRPV4), regulate the function of the periodontal ligament (PDL) of the DAJ [7, 8] as well as cartilage and subchondral bone in other diarthrodial joints, such as the knee [9, 11, 13, 16, 19, 23, 26, 29, 31]. In our previous study, we found that elevated expressions of MS-ion channels in response to compressive (PIEZO1) and tensile (TRPV4) strains regulated PDL cell homeostasis through the influx and efflux of biometal zinc (Zn²⁺), influencing mitochondrial function (MFN2), oxidative state (hypoxia inducible factor-1α (HIF-1α)), and cell senescence (p16) [30]. Although numerous studies report that abnormal masticatory loads can affect TMJ function [6], the mechanisms underlying load-mediated TMJOA remain unclear. If and how manipulation of mastication loads can regenerate tissues and lead to recovery of abnormal load-induced damage are less investigated. We will detail the effects of recovering a hypofunctioning TMJ using normal bite forces by **(1) **mapping the biomolecular differential expressions in the inner and outer layers of a rat TMJ cartilage, and **(2)** correlate with physicochemical characteristics of the condyles. We will detail the activation of MS-ion channels PIEZO1 and TRPV4 and their downstream effects, including Zn^2+^ homeostasis and cell metabolism, using complementary models in vivo and in vitro.

## Materials and methods

The University of California San Francisco (UCSF) Institutional Animal Care and Use Committee approved the experimental procedures used in this study (approval no. IACUC protocol AN200023-00I) on February 11, 2025.

### In vivo and in vitro experimental workflows (Fig. 1)

**Fig. 1:**
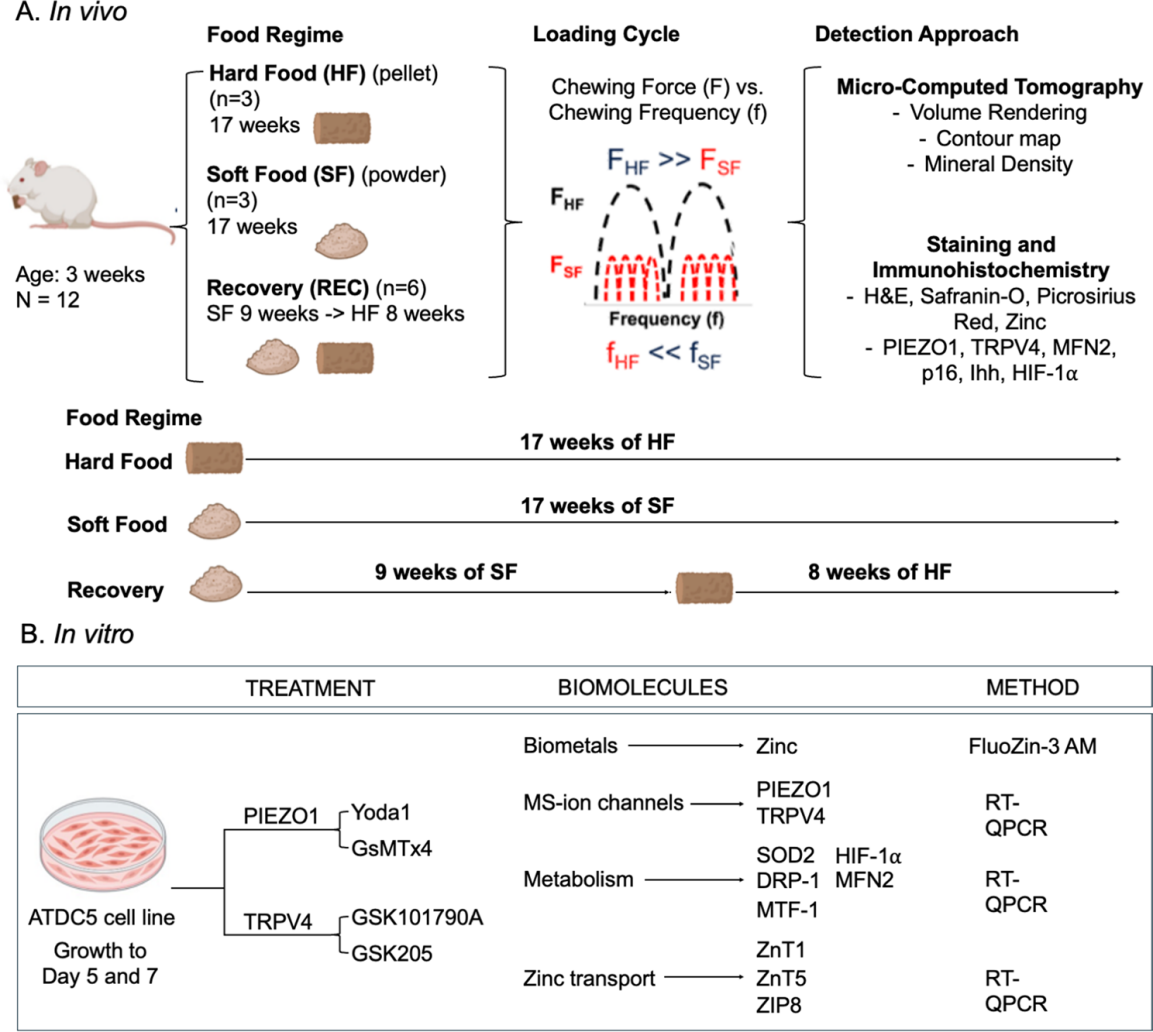
Experimental Design: Investigating bite forces and mechanobiology in TMJ and chondrocytes using both animal (in vivo) and alternate models (in vitro). **(A)** Twelve male Sprague-Dawley rats received hard-food (HF, *N* = 3) and soft-food (SF, *N* = 3), and a third group, the recovery (REC, *N = 6*) was given 9 weeks of SF and was immediately switched to 8 weeks of HF Theoretical projection of reactionary loading from the dentoalveolar joint to the temporomandibular joint in the rats from HF, SF, and Rec groups are shown. The condyles from the temporomandibular joints (TMJs) were prepared for CT imaging, histology, and immunohistochemistry. **(B)** The ATDC5 chondrocyte behavior at 5 days ((D5), *N* = 12) and 7 days ((D7), *N* = 12) following treatments with agonists of PIEZO1 (Yoda1, *N* = 6) and TRPV4 (GSK101790A, *N* = 6), and antagonists of PIEZO1 (GsMTx4, *N* = 6) and TRPV4 (GSK205, *N* = 6) for 2 h were mapped. Quantitative analyses of biometal zinc (Zn), and biomolecules PIEZO1, TRPV4 (MS-ion channels), SOD2, DRP-1, MTF-1, HIF-1α, and MFN2 (metabolism), ZnT1, ZnT5, and ZIP8 (Zn transporters) was performed.

### In vivo workflow

Twelve male Sprague-Dawley rats were divided into three groups, hard (HF), soft (SF), and recovery (REC). Each group received a specific food regimen. Foods included, hard-food (HF, *N* = 3) and soft-food (SF, *N* = 3). Hard-food constituted pellets, soft-food was powdered hard-food (PicoLab Rodent Diet 20 5053, LabDiet, St, Louis, MO) [18]. The REC group (*N* = 6) was given 9 weeks of SF and was immediately switched to 8 weeks of HF. Rats were euthanized at 17 weeks (IACUC protocol AN200023-00I, UCSF) and animals were imaged using a preclinical CT, followed by dissection of the condyles for histology.

### In vitro workflow

The ATDC5 cell line was utilized as an in vitro model to investigate chondrocyte behavior at 5 days ((D5), *N* = 12) and 7 days ((D7), *N* = 12). Analogous to loading schemes in vivo, cells were mechanically activated with agonist of PIEZO1 (Yoda1, *N* = 6), agonist of TRPV4 (GSK101790A, *N* = 6), and blunted with antagonist of PIEZO1 (GsMTx4, *N* = 6), or antagonist of TRPV4 (GSK205, *N* = 6) for 2 h (Table S3). Zn staining was performed with FluoZin-3 AM alongside reverse-transcription quantitative PCR (RT-qPCR) for quantification of biomarkers, PIEZO1 and TRPV4 (MS-ion channels), SOD2, DRP-1, MTF-1, HIF-1⍺, and MFN2 (metabolism), ZnT1, ZnT5, and ZIP8 (Zn transporters) (Table S4).

### Calibrated preclinical X-ray computed tomography on condyles

The digital volumes of euthanized animals imaged using X-ray CT at an energy of 50 kV were used to evaluate bone volume fraction (BVF, bone volume/(total volume = pore volume + bone volume)) and morphology of the condylar surface and topology. The bone mineral densities (BMD) in µg/cm^3^ of left and right condyles from all three groups were evaluated. Associations between BMD and BVF depth profiles from condylar surface into bone within and across all three groups were mapped.

### Cartilage thickness and porosity profiles from cartilage through subchondral regions into bone

The fixed (10%NBF) and decalcified (20% EDTA) 5 μm thick sections of condyles were individually stained with Hematoxylin and Eosin (H&E), Safranin-O (Saf-O) countered with Fast Green, and Picrosirius Red (PSR) and imaged using a light microscope (AXIO-Z3, Zeiss Microscopy, Pleasanton, CA). Cartilage thickness was measured using Saf-O stained tissue sections at fifty locations per image using ImageJ. Tissue sections comparable to tomograms were also used to estimate changes in trabecular space from condylar surface into bone using skeletalization function (AVIZO 3D, 2024.1, Thermofisher, Waltham, MA). The peak positions and full width at half maximum (FWHM) representative of depth-specific trabecular space were estimated using Gaussian distribution.

### Immunolocalized biomolecules in vivo

Serial sections were immunolocalized for antigens TRPV4 (1:200), PIEZO1 (1:50), mitofusin2 (MFN2, 1:200), p16 (1:100); Indian hedgehog (Ihh, 1:100), hypoxia-inducible factor-1α (HIF-1α, 1:200). Sections were counterstained with hematoxylin (immunohistochemistry-IHC) or DAPI (immunofluorescence-IF). Three sections per animal per group were imaged using a light microscope (AXIO-Z3, Zeiss Microscopy, Pleasanton, CA), and quantitative spatial maps of immunolocalized biomolecules were generated using ImageJ [22].

### Agonist and antagonist treatments of ATDC5 MS-ion channels, intracellular zinc, and real-time quantitative reverse transcription PCR

Chondrogenic mouse cells (ATDC5, Riken Cell Bank, Tsukuba, Japan) were grown to day 3 (D3, proliferative), day 5 (D5, proliferative), and day 7 (D7, hypertrophic) in DMEM/F12 medium in a cell incubator at 37°C and 5% CO_2_. For cells grown to D7, 50 µg/mL ascorbic acid and 10mM β-glycerophosphate were added at D5 to differentiate cells from proliferative to hypertrophic chondrocytes. At each time point, cells were treated with Yoda1 (PIEZO1 agonist), GsMTx4 (PIEZO1 antagonist), GSK1016790A (TRPV4 agonist), and GSK205 (TRPV4 antagonist) for 2 h. Effective dosage for each treatment were verified using the CyQUANT™ MTT Cell Viability Assay (Thermo Fisher Scientific, Waltham, MA) (Table S3 Figure S2).

Treated cells were fixed with 4% PFA/PBS and incubated for 2 h with 3µM of FluoZin^TM^-3 (Thermo Fisher Scientific, Waltham, MA) to detect intracellular Zn. Cells were mounted and counterstained using ProLong™ Gold Antifade Mountant with DAPI (Thermo Fisher Scientific, Waltham, MA) and imaged using a Zeiss microscope (AxioObserver-2, Zeiss Microscopy, Pleasanton, CA). Corrected total cell fluorescence was calculated in ImageJ for five images per treatment.

RNA from each time point was also extracted and processed for real-time quantitative reverse transcription PCR (RT-q-RT-PCR) using a RNeasy Mini Kit (QIAGEN, Redwood City, CA). Gene expressions of PIEZO1, TRPV4, ZIP8, ZnT1, ZnT5, HIF-1⍺, SOD-2, DRP-1, MTF1, and MFN-2 were determined using primers (Table S4).

### Statistical analysis

#### Spearman correlation and hierarchical clustering of outcomes from in vivo and in vitro models

Stratifying by HF/SF/REC status (group) for in vivo replicates and agonists/antagonists status (group) for in vitro replicates, biomarkers/metals were clustered by their averaged expression across their IL/OL regions, and Spearman’s correlation matrices were calculated to determine statistical meansure of biomolecular/biometal associations. The PIEZO1/TRPV4 agonists and antagonists were separately clustered globally across all in vitro specimens using hierarchical mapping.

For in vitro experiments, observations varied by agonist and antagonist treatments and age of cells. Hierarchical maps were created from normalized RT-qRT-PCR data and Zn expressions and rescaled to illustrate clustering and associations between biomarkers and specimens. Hierarchical clustering was performed independently for D5 and D7 for in vitro experiments, to note if the markers clustered by biological pathway on respective days. 

#### Physical properties and BMD analyses of left and right condyles

Unpaired Student’s t-test with a P-value < 0.05 was used to identify statistical significance in BVF, morphology, and BMD across all groups in vivo, and agonist and antagonist groups in vitro.

## Results

### Expressions of MS-ion channels, cell metabolism, and cell fate in a hypofunctioning TMJ can be altered by switching the bite forces from soft to hard foods

**Fig. 2:**
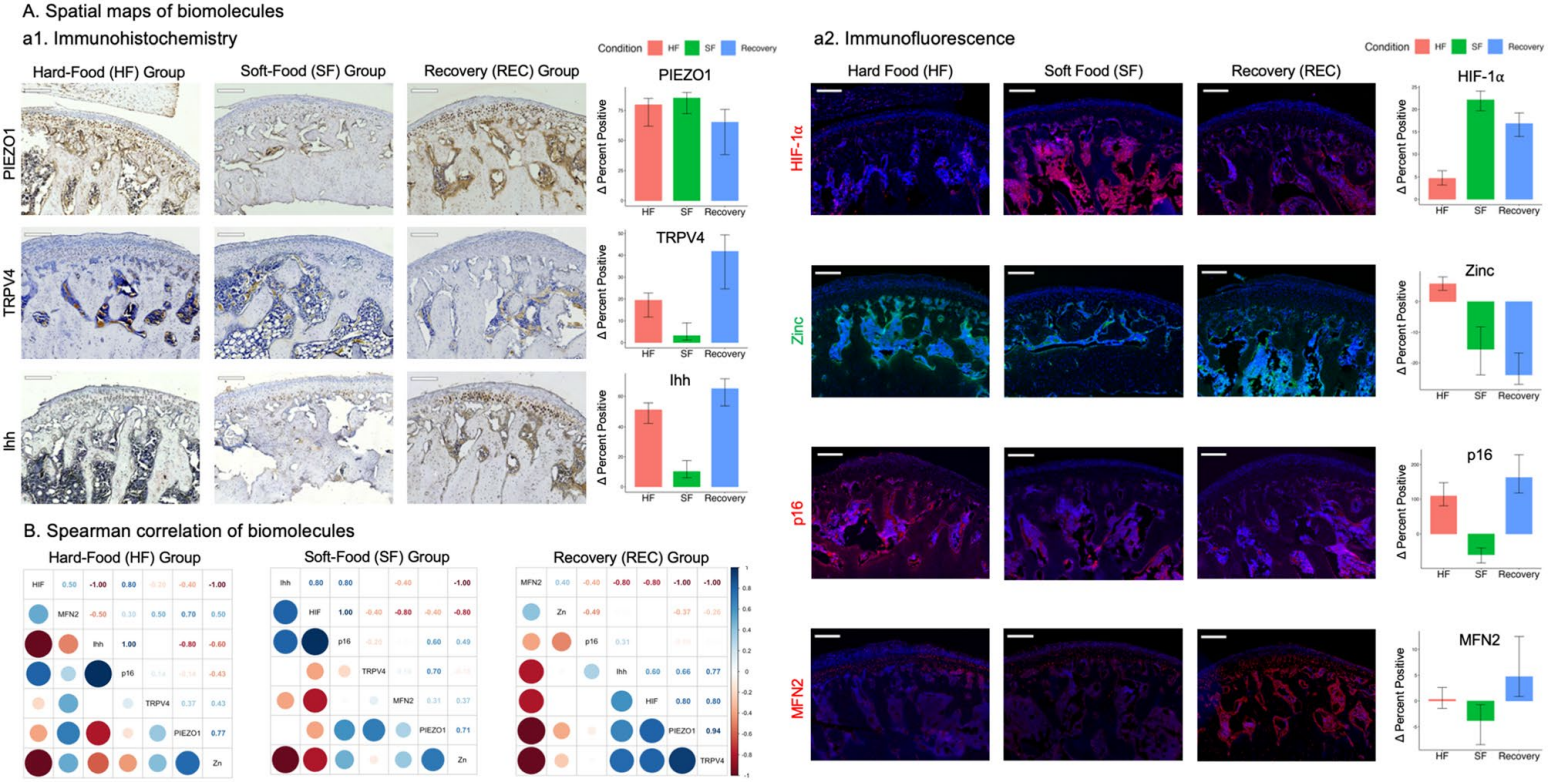
Differential expressions of PIEZO1 and TRPV4 in TMJ cartilage and bone under different bite forces. **(A)** PIEZO1 was expressed in the fibrous outer layer (OL) of HF, and was not observed in SF and REC. On the contrary, TRPV4 was expressed in the IL (subchondral) and bone regions of the HF, SF, and REC. PIEZO1 and TRPV4 were expressed in the OL (proliferative zone) of SF and REC compared to HF. In contrast, PIEZO1 and TRPV4 were expressed in the IL (hypertrophic chondrocytes and subchondral bones) of HF and REC groups. SF animals had significantly lower proliferation than HF, determined by a decrease in expression of the proliferative marker Indian hedgehog (Ihh), increase in cell senescence marked by p16, and decrease in metabolism marked by MFN2. The REC group displayed similar proliferation to HF (Ihh), decreased cell senescence (p16 stats), and an increase in metabolism (MFN2). HIF-1⍺ and Zn were decreased in the SF group but restored in the REC group. Counter-stained by hematoxylin (DAB staining, **a1**) or DAPI (immunofluorescence, **a2**). Scale bar = 200 μm. **(B)** Spearman correlation illustrates that Zn is positively correlated with PIEZO1 and TRPV4, and MFN2 and negatively correlated with HIF-1α, Ihh, and p16. However, in SF, Zn is positively correlated with PIEZO1, MFN2 and p16, and continues to be negatively correlated with HIF-1α and Ihh. In REC, Zn is positively associated only with MFN2, negatively associated with p16 and the MS-ion channels, and has no association with HIF-1α and Ihh. Data are presented as median, IQR, and 95% CI for bar charts or mean (SD) otherwise. A significant increase (+ ve) or decrease (-ve) with a *p* < 0.05 are presented in Table 1. Scale bars: 200 μm

Data from rats are presented as median, IQR, and 95% CI for bar charts or mean (SD) unless stated otherwise. The differences (Δ) in fold expression change and odds ratio (FC/OR) (Y-axis of the barcharts) between inner and outer layers (IL, OL) across groups are shown (Table 1, S1, S2). The PIEZO1 of HF was significantly greater than SF and REC (*p* < 0.001, *p* < 0.105) (Table S1). In the OL of the TMJ from HF group, the MS-ion channel PIEZO1 was expressed in cells within the fibrous layer and in the proliferating chondrocytes underneath (Figs. 1A, a1; S1), while a few TRPV4 positive cells were observed in the OL (Figs. 2A, a1; S1). In the IL of the TMJ from HF group, both PIEZO1 and TRPV4 were expressed in the hypertrophic chondrocytes and bone cells of the subchondral bone. SF significantly decreased PIEZO1 and TRPV4 expressions in OL and IL. Interestingly, in REC group, no PIEZO1 positive cells were observed in the OL. Similarly, decreased expressions of the chondrocyte proliferation/differentiation marker Ihh (*p* < 0.001) were observed in SF, and indicated a significantly lower chondrocyte proliferation/differentiation in SF compared to HF (Figs. 2A, a1; S1). These changes in SF were accompanied with an increase in p16 (*p* = 0.028) and decrease in MFN2 (*p* = 0.001) (Figs. 2A, a2; S1). The Ihh in REC group was significantly different from HF (Ihh) (*p*= 0.001), but decreased p16 (*p* = 0.016), with no increase in metabolism (MFN2) (*p* = 0.994). In addition, HIF-1⍺ (*p* = 0.001) and Zn (*p* = 0.001) were decreased in SF group but restored in REC (*p* = 0.001, *p* = 0.001) group (Figs. 2A, a2; S1, Table 1, S1, S2).

**Table 1:**
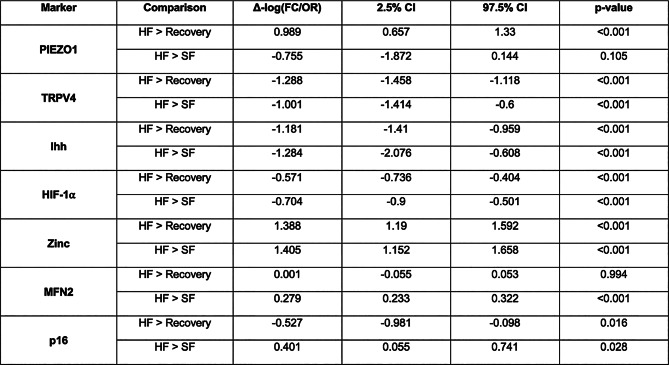
Differences (Δ) between conditions in log-fold change / odds ratios representing relative differences in expression / positivity between inner and outer regions, with hard food as a reference group

These data are summarized through a Spearman correlation plot included in Fig. 2. In HF group, Spearman correlation illustrated that Zn is positively correlated with PIEZO1 and TRPV4, and MFN2, and negatively correlated with HIF-1α, Ihh, and p16 (Fig. 2B). However, in SF, Zn is positively correlated with PIEZO1, MFN2 and p16, and negatively correlated with HIF-1α and Ihh. In REC, Zn is positively associated only with MFN2, negatively associated with p16 and the MS-ion channels, and has no association with HIF-1α and Ihh (Fig. 2B).

### Blunting of PIEZO1 and TRPV4 alters Zn homeostasis and cell metabolism in vitro

**Fig. 3:**
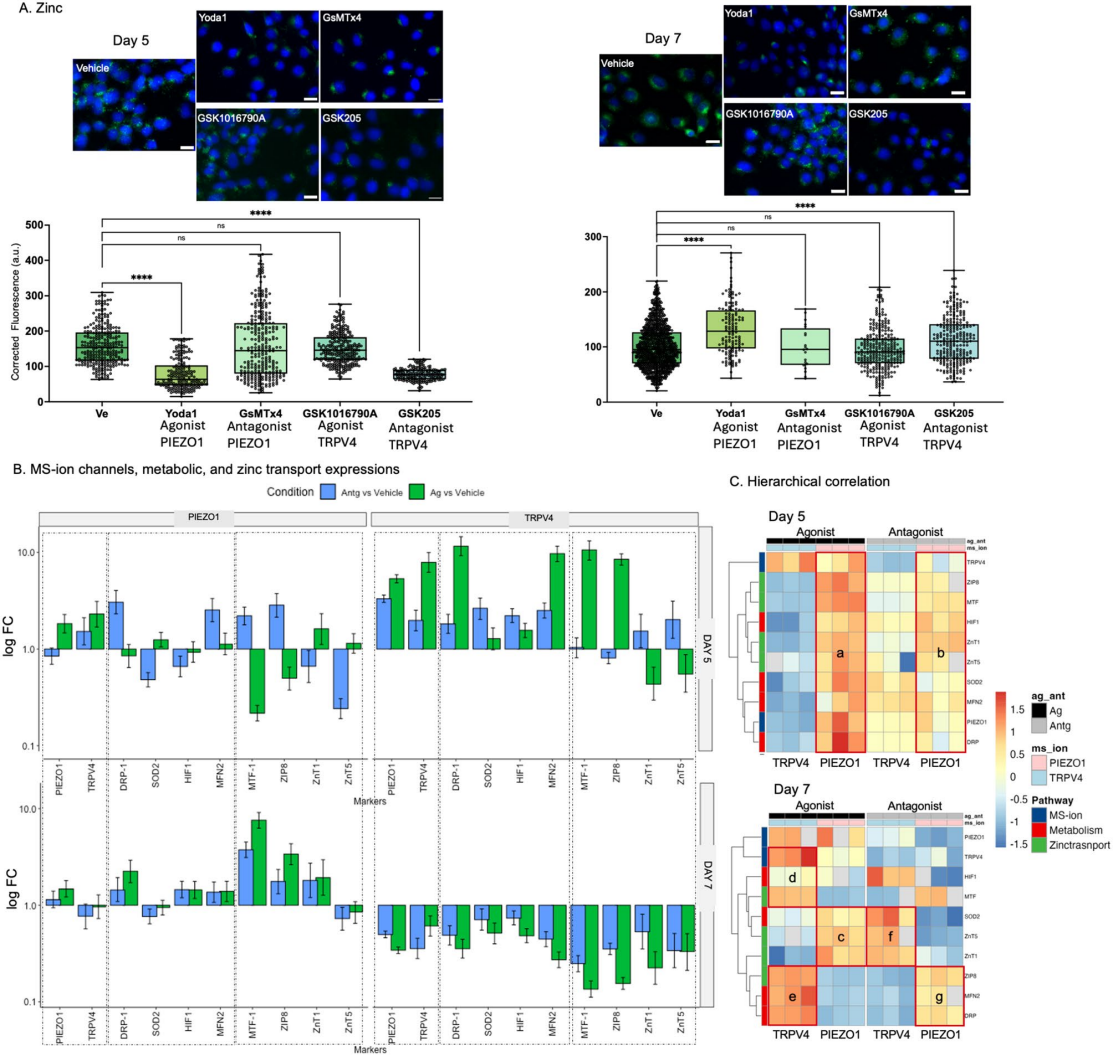
PIEZO1 and TRPV4 agonists and antagonists influenced Zn levels and temporal changes in gene expression in ZnTs, and cellular metabolism. **(A)** At D5 and D7, PIEZO1 and TRPV4 channels were activated (an increase relative to vehicle) or blunted (a decrease relative to vehicle) by PIEZO1 (Yoda1, GSK1016790A) and TRPV4 (GsMTx4, GSK205) agonists and antagonists respectively. Zn levels decreased between vehicle and cells treated with PIEZO1 agonist and TRPV4 antagonist at D5 and increased at D7. No significant differences were observed between vehicle, and PIEZO1 antagonist and TRPV4 agonist at D5 and D7. Scale bar − 20 μm. **(B)** The logFC of gene expressions with PIEZO1 agonist and antagonist at D5 and D7 were of heterogenous relationships, while for TRPV4 agonist and antagonist were consistent with an increase in D5 and a decrease in D7. **(C)** Hiearchical correlations indicated increased expressions of all transcripts with PIEZO1 agonist at D5 (box a) and to a lesser extent with antagonist (box b). However, at D7 the increased expressions of SOD2, and ZnT1 and ZnT5 remained steady (box c) with PIEZO1 agonist and decreased with antagonist (box d). With TRPV4 agonist, at D7, an increased expression in MTF1 with HIF-1α (box d), ZIP8, DRP, and MFN2 (box e), and to a lesser extent SOD2 were observed. It appears that with an increase in load (agonist) that there is a concomitant PIEZO1 and TRPV4 axes development from D5 through D7 with a decrease in HIF-1α in D7. The two axes include TRPV4-MTF1-MFN2-DRP-ZIP8 axis where intracellular Zn is controlled by ZIP8, and PIEZO1-SOD2-ZnT5 axis with an increase in Zn levels controlled by ZnT5. A decrease in load however encourages a flipped axes with the MSion channels in that TRPV4-SOD2-ZnT1/5, and PIEZO1-MFN2-DRP-ZIP8 were observed. **Note**: D5 and D7 – at Day 5 and Day 7; Log fold change (logFC) is the ratio of biomolecular expression levels in test condition (treated specimen) compared to control (vehicle). Scale bars: 20 μm

The agonists and antagonists-specific (Table S3) MTT assays on ATDC5 cells in vitro provided safe dose (red dots and arrows, Fig. S2). At D5 and D7, PIEZO1 and TRPV4 channels were activated (an increase relative to vehicle) or blunted (a decrease relative to vehicle) by PIEZO1 (Yoda1, GSK1016790A) and TRPV4 (GsMTx4, GSK205) agonists and antagonists respectively. Zn levels decreased between vehicle and cells treated with PIEZO1 agonist and TRPV4 antagonist at D5 and increased at D7. No significant differences were observed between vehicle, and PIEZO1 antagonist and TRPV4 agonist at D5 and D7 (Fig. 3A). Figure 3B give us the relative differences expressed as a fold change between agonist and antagonist with respect to the vehicle. However, Figure S4 provides relative expressions of biomolecules from ATDC5 cells from vehicle, agonist, and antagonist groups in log scale. The relative differences with vehicle in log(FC) at days 5 and 7 and significane values are shown in Table S6.

As shown in Fig. 3, ZnT5 expression was different from D5 to D7. An decreased expression of SOD2 in D5 compared to D7 specifically after ascorbic acid treatment was observed (Fig. 3B, Fig. S4, Tables S5, S6). Hierarchical clustering was performed independently for D5 and D7 for in vitro experiments using data shown in Figure S4, to note if the markers clustered by biological pathway on respective days. Increased expressions of all transcripts were observed with PIEZO1 agonist at D5 (box a) and to a lesser extent with antagonist (box b). However, at D7 the increased expressions of SOD2, and ZnT1/5 remained steady (box c) with PIEZO1 agonist and decreased with antagonist (box d). With TRPV4 agonist, at D7, an increased expression in MTF1 with HIF-1α (box d), ZIP8, DRP, and MFN2 (box e), and to a lesser extent SOD2 were observed. With load increase (agonist) there is a concomitant PIEZO1 and TRPV4 axes development from D5 through D7 with a decrease in HIF-1α in D7, encouraging TRPV4-MTF1-MFN2-DRP-ZIP8 axis where Zn levels are controlled by ZIP8, with PIEZO1-SOD2-ZnT5 axis and an increase in Zn levels are controlled by ZnT5. A decrease in load (antagonist) however encouraged a flipped axes with the MSion channels in that TRPV4 correlated with SOD2-ZnT1/5, and PIEZO1 with MFN2-DRP-ZIP8 (Fig. 3C, Tables S5, S6) indicating the effect of reduced load on Zn levels and cell metabolism.

### Hard diet regulated bite forces alter hypofunctioning condylar physical properties, mineral densities, and morphology

**Fig. 4:**
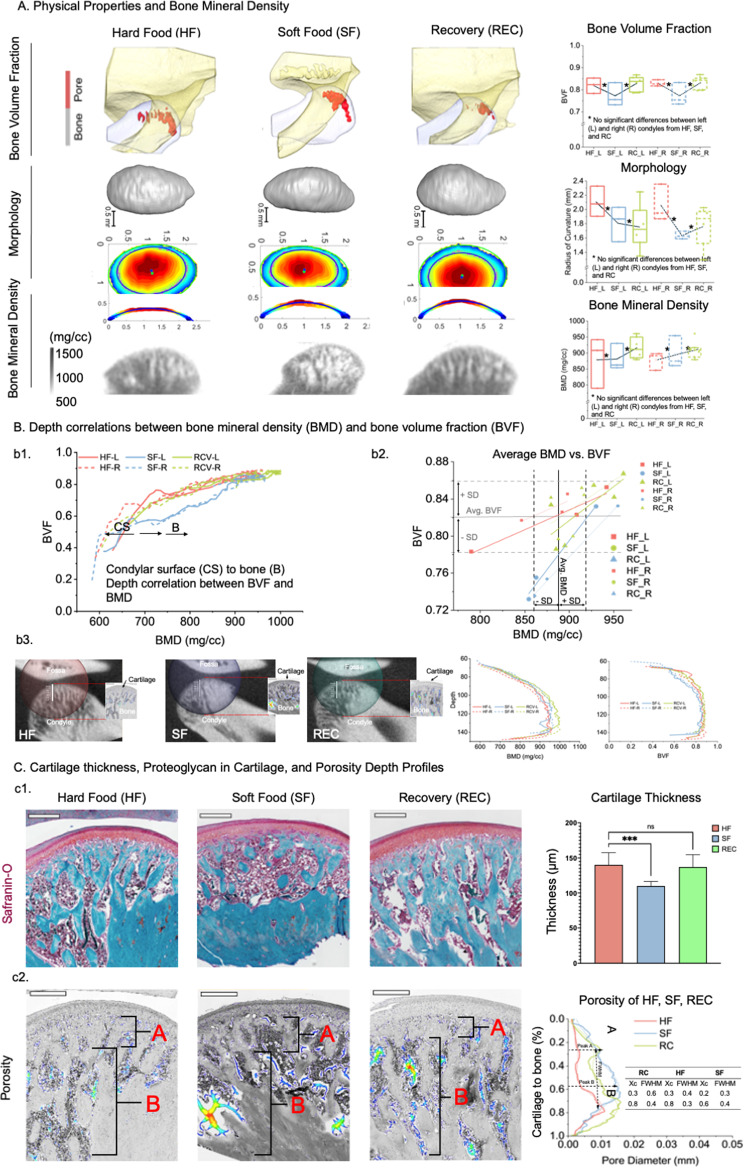
The physical properties and bone mineral density of REC trended toward healthy levels compared to SF. **(A)**. A decrease in bone volume fraction (BVF) and condylar morphology (radius of curvature) and bone mineral density (BMD) were observed in SF contrasting an increase in REC. Physical properties of SF diverged from HF, but REC converged to HF (bone volume fraction (BVF)), and condylar morphology (radius of curvature)) and bone mineral density (BMD)). **(B)**. Depth correlations between BMD and BVF within and across all groups are shown in (b1). Average BMD vs. BVF of REC converged with HF, but SF group diverged from HF. Standard deviation (SD) and average BMD and BVF also are plotted (b2). Histology sections shown in C are correlated with X-ray intensity maps of the same condyle/rat within each group to illustrate BMD variations. **(C)**. Safranin-O/Fast Green stain (organ/red tissue above blue bone) illustrated a significantly decreased cartilage thickness in SF (*P* < 0.001) compared to no difference in REC relative to HF (not significant - ns) (c1). Porosity depth profiles indicated two significant peaks. Peak A closer to cartilage, and peak B in bone. Peaks A and B were of similar depths in HF and REC, but peak B was closer to SF condylar surface (c2). Scale bars: 400 µm

In our in vivo model, at a tissue level, a decrease in bone volume fraction (BVF, first row, Fig. 4A) and condylar morphology (radius of curvature, second row, Fig. 4A) and bone mineral density (BMD, third row, Fig. 4A) were observed in SF group. This decreasing trend contrasted with an increasing trend of the same properties but in REC group (Fig. 4A). Physical properties of SF diverged from HF, but REC converged to HF (Fig. 4A). A switch in bite forces from soft to hard restored the physical properties of REC group compared to HF group.

Depth profiles of BVF associated with BMD from the condylar surface (CS) to bone (B) were similar for all groups with REC group in between HF and SF groups both for the left and right condyles (**b1**, Fig. 4B). However, the change in BMD vs. BVF trended the same for REC as HF, but was steeper for SF group (**b2**, Fig. 4B) indicating an imbalance between formation and resorption events in SF compared to a regain of this balance in REC group. H&E (Fig. S5) and Safranin-O/Fast Green stain (orange/red tissue above blue bone) illustrated a significantly decreased cartilage thickness in SF (*P* < 0.001) and collagen changes (PSR staining, Fig. S5, bottom row) in SF, as well as a restoration in REC relative to HF (not significant-ns) (**c1**). Porosity depth profiles indicated two significant peaks; peak A closer to cartilage, and peak B in bone, and position of these peaks were of similar depths in HF and REC, but peak B was closer to condylar surface in SF (**c2**).

## Discussion

Mastication or chewing-induced compression influences deformation related mechanical strains (solid strains) within the OL and IL of cartilage and trabecular bone of the TMJ condyle. The resulting mechanoactivation of layer-specific metabolism is governed by homeostasis of chondrocytes and bone cells within. PIEZO1 and TRPV4 in HF cartilage expressed layer-specific patterns in that PIEZO1 was observed in progenitor chondrocytes in OL proliferative zone and in IL hypertrophic chondrocytes of subchondral zone. In contrast, TRPV4 was expressed only in IL hypertrophic chondrocytes (Fig. 2A; Table 1, S1,S2). This suggested HF-induced compressive stress from the condylar surface regulated PIEZO1 expression and, in turn, mediated early-stage chondrocyte differentiation. Reduced mastication loads by SF blunted the two MS-ion channels in cells at all stages, affected layer-specific metabolism (Fig. 3, Tables S5, S6), and plausibly induced morphological changes in cartilage and subchondral bone thickness (Fig. 4). In contrast, restoring the mastication loads in REC group induced a compensatory activation (higher expression than the control/HF TMJ) of PIEZO1 and TRPV4 in all stages of chondrocytes, and led to a “catch up” of matrices within the outer and inner layers, and trabecular bone formation.

Similar to the DAJ [30], the blunted MS-ion channels in SF group also affected Zn production in the hypertrophic chondrocytes at the cartilage-bone interface in the TMJ (Fig. 2B). These results corroborated with temporal evaluation of ATDC5 cells. PIEZO1 and TRPV4 agonist or antagonist treatments to ATDC5 cells affected their intracellular Zn levels via MTF-1, Zn import via ZIP8, Zn export via ZnT1 and ZnT5 [10]. Like bone, the upstream stimulus such as mechanical load on the TMJ cartilage is essential to mediate the downstream chondrocyte metabolism. Zn homeostasis is critical for maintaining mitochondrial function and cycle [5]. SF blunted MS-ion PIEZO1 and TRPV4 (Fig. 2A, a1), decreased mitochondrial fusion (MFN2), and increased mitochondrial fission (DRP-1) (Fig. 3B), resulting in a fragmented mitochondrial network/cycle and reduced mitochondrial number in the chondrocytes. These trends observed in pre-hypertrophic and hypertrophic chondrocytes (Fig. 2A, a2), resulted in mitochondria dysfunction. Abnormal mitochondrial function induced hypoxia and abnormal oxidative states of chondrocytes, finally led to ROS as indicated by SOD2 and p16 (Figs. 2A-a and 3B 2). Taken together, reduced mastication loads (SF) blunted MS-ion channels PIEZO1 and TRPV4 in chondrocytes, and resulted in abnormal chondrocyte metabolism.

**Fig. 5:**
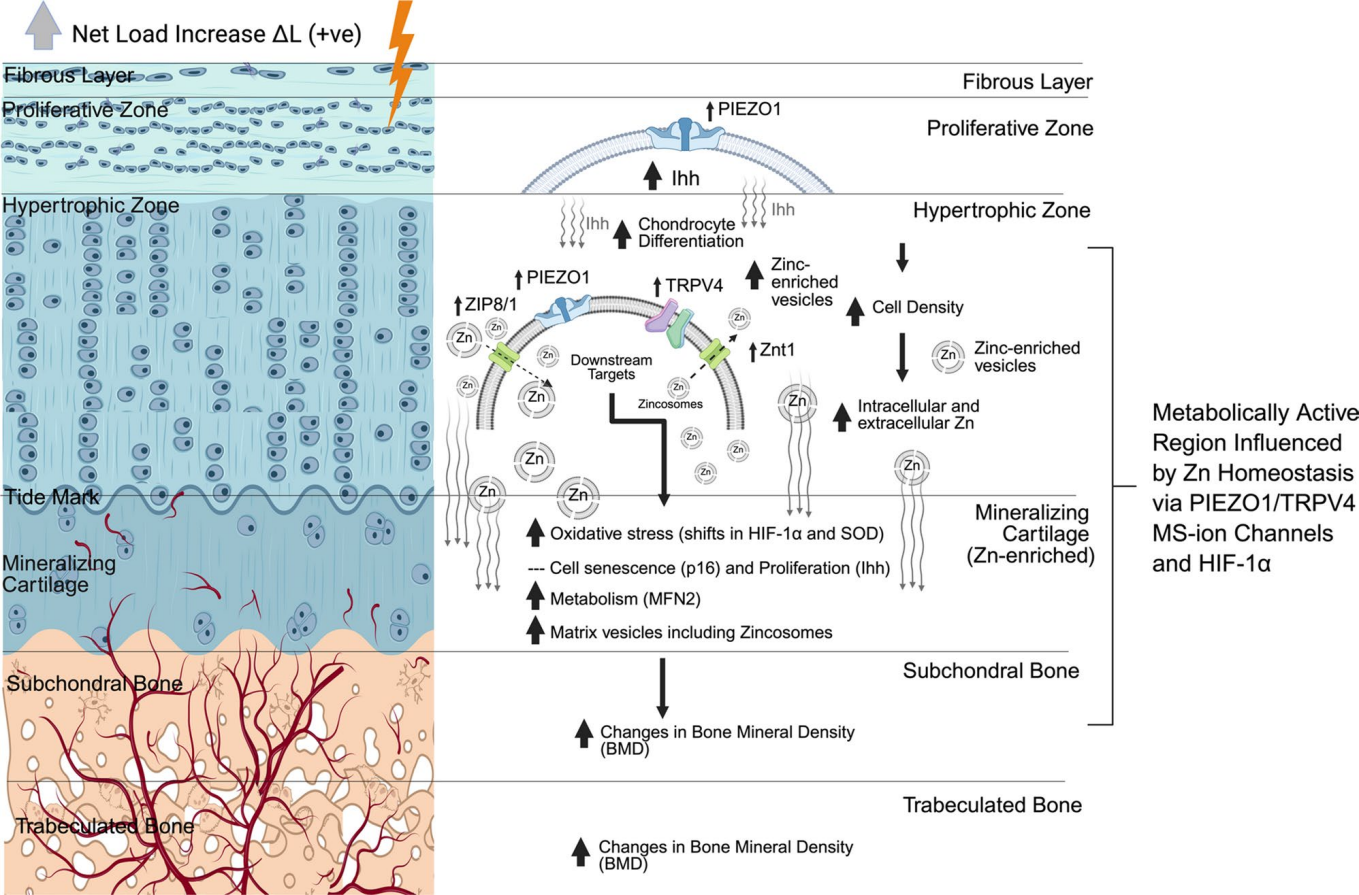
Working Hypothesis - Bite force restoration activates mechanosensitive pathways and Zn signaling associated with TMJ cartilage and bone recovery through MS-ion channel activities and HIF-1α related cellular responses. The recovery of a hypo-functioning TMJ by switching the bite forces from SF to HF (ΔL(+ve); grey block arrow) will induced deformation in cartilage, bone, and the hypertrophic cartilage-subchondral bone interface, and activate PIEZO1 and TRPV4 Ms-ion channels in tissue and zone-specific cells. Activation of MS-ion channels will induce mechanotransduction and alter Zn levels and flow of Zn through Zn transporters (ZnT1/T5) and regulate Ihh to stimulate differentiation of proliferating cells into chondrocytes and maintain normal chondrocyte density in the cartilage. These cell responses maintain normal/low oxidative (HIF-1α) and ROS production (SOD2), increase/maintain mitochondrial function (MFN2) and cell metabolism, cell fate (p16), and recover the subchondral bone volume (BV/TV) and mineral density (BMD).

Chondrocyte differentiation depends on normal cell metabolism [25]. Abnormal chondrocyte metabolism in SF rats reduced Ihh in proliferating/hypertrophic chondrocytes and decreased chondrocyte proliferation and differentiation but induced chondrocyte senescence in OL. This mechanically regulated biological cascade by virtue of changing bite forces decreased hypertrophic chondrocytes in IL and changed the hypertrophic zone and cartilage widths but with a loss in matrix structure (Saf-O/Fast Green, PSR stains) (Figs. 4, S5). Macromolecules PGs and collagen provide compression resistance to cartilage [14], and their loss due to hypofunction (SF instead of HF) altered tissue response, affected mineralizing subchondral bone, and induced bone loss (Fig. 4). Decreased trabecular bone volume and bone mineral density may be due to direct effects of lower mastication loads resulting in a decreased number of hypertrophic chondrocytes, and less transdifferentiation of chondrocytes into osteoblasts at the cartilage-bone boundary, lower matrix production, and decreased mineralization. However, no significant differences between left and right condyles within each group revealed bilateral homogeneity (Fig. 4). This is because, preferential chewing noted in humans [15, 21] does not exist in rats [17]. Regardless, a significant increase in chondrocyte metabolic markers by recovering the chewing forces to HF from SF was observed. This change in load from hypofunction to regular function also limited cell senescence (p16) resumed chondrocyte differentiation (Ihh) and increased number of chondrocytes and cartilage matrix production (Figs. 2 and S5) with some biomarkers expressions in REC at higher levels than control/HF group suggesting a load-mediated compensatory effect. The BMD and BVF also significantly improved (Fig. 4A and B). From this study, we hypothesize that morphological and BMD changes in tissue/zone-specific TMJ regions could be regulated by Zn homeostasis, metabolism, cell differentiation by stimulating PIEZO1 and TRPV4 mechanosensory channels in cells that also are HIF-1α positive and region-specific within a condyle stimulated by prescribed mechanical loads (Fig. 5).

Study limitations seen through inconsistent in vitro and in vivo outcomes are due to different experimental conditions. The evidence of Zn positive nodules in ATDC5 cells corroborated with previous data from our laboratory on nodules during endochondral ossification [28]. Future studies are warranted to map the role of these region-specific Zn positive nodules and their concentration effect on mineralization [12], that is, the effect of the switch in magnitude and frequency of mastication loads on chondrocyte to osteoblast transdifferentiation and the functional evaluation of subchondral and trabecular bones. Future studies should include extended mixed mode prescription of chewing forces (SF and HF), a practical outcome with a translational output that could combat tissue degeneration.

## Supplementary Information

Below is the link to the electronic supplementary material.

Supplementary Material 1

## Data Availability

No datasets were generated or analysed during the current study.

## Nomenclature

BMD: Bone mineral density
BVF: Bone volume fraction
CT: Computed tomography
DAJ: Dentoalveolar joint
DRP-1: Dynamin related protein-1
D5 and D7: Days 5 and 7
FWHM: Full width at half maximum
HIF-1α: Hypoxia inducible factor–1α
HF: Hard–food
Ihh: Indian hedgehog
IHC: Immunohistochemistry
IF: Immunofluorescence
IL: Inner layer
Log(FC): Log fold expression change
MS: Ion–mechanical sensory ion channel
MTF–1: Metal–regulatory transcription factor 1
MFN2: Mitofusin 2
OL: Outer layer
p16: Cell senescence
PDL: Periodontal ligament
PSR: Picrosirius Red
RT: qPCR–reverse–transcription quantitative PCR
REC: Recovery group
SOD2: Superoxide dismutase 2
SF: Soft–food
Saf-O: Safranin-O and Fast Green
TMJ: Temporomandibular joint
TMJOA: Temoporomandibular joint osteoarthritis
TRPV4: Transparent Receptor Potential Cation Channel Subfamily V Member 4
Zn: Zinc
ZnT1, ZnT5, ZIP8: Zinc transporter 1 and 5, and 8
