# Supplementary material for: Chewing loads alter TMJ metabolism and Zn homeostasis via PIEZO1/ TRPV4
Source: J Biol Eng. 2025 Nov 28;20:1. doi: 10.1186/s13036-025-00564-2 (PMC12764147; doi:10.1186/s13036-025-00564-2)
Supplement: Supplementary file 1 — Supplementary Material 1 [file 13036_2025_564_MOESM1_ESM.pdf]

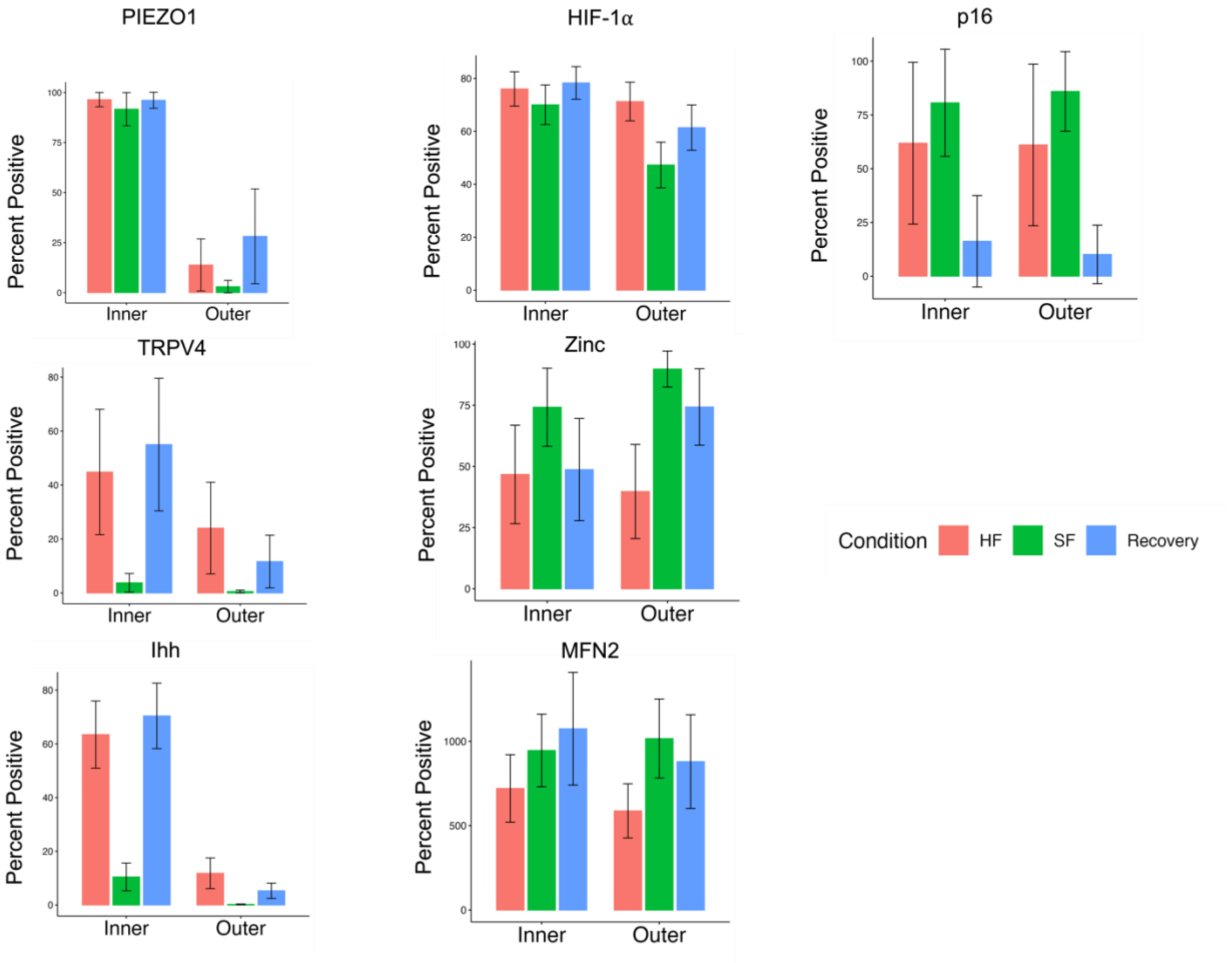

Figure S1. Region-specific biomolecular expressions: Percent positive cells expressing specific biomolecules in the inner and outer zones of the condyles from HF (red), SF (blue), and REC (green) groups.

Marker20	Comparison	Region	log(FC/OR)	2.5% CI	97.5% CI	p-value
PIEZO1	HF > Recovery	Inner	0.13	-3.41	3.29	0.94
	HF > SF	Inner	0.83	-2.61	4.10	0.621
	Recovery > SF	Inner	0.81	-3.06	4.53	0.656
	HF > Recovery	Outer	-0.87	-4.39	2.28	0.542
	HF > SF	Outer	1.63	-1.85	4.94	0.358
	Recovery > SF	Outer	2.54	-1.19	6.41	0.166
	Inner > Outer	HF	5.13	4.87	5.40	<0.001
	Inner > Outer	Recovery	4.14	3.93	4.34	<0.001
	Inner > Outer	SF	5.88	5.05	6.94	<0.001
TRPV4	HF > Recovery	Inner	-0.43	-3.52	2.43	0.743
	HF > SF	Inner	3.03	-0.34	5.75	0.079
	Recovery > SF	Inner	3.42	0.03	6.66	0.049
	HF > Recovery	Outer	0.85	-2.18	3.70	0.506
	HF > SF	Outer	4.03	0.64	6.77	0.022
	Recovery > SF	Outer	3.13	-0.25	6.46	0.069
	Inner > Outer	HF	0.94	0.83	1.06	<0.001
	Inner > Outer	Recovery	2.23	2.11	2.36	<0.001
	Inner > Outer	SF	1.94	1.57	2.34	<0.001
Ihh	HF > Recovery	Inner	-0.31	-2.07	1.38	0.678
	HF > SF	Inner	2.70	0.79	4.38	0.012
	Recovery > SF	Inner	3.04	1.03	4.79	0.01
	HF > Recovery	Outer	0.87	-0.90	2.57	0.304
	HF > SF	Outer	3.99	2.00	5.79	0.002
	Recovery > SF	Outer	3.14	1.04	5.09	0.008
	Inner > Outer	HF	2.57	2.42	2.72	<0.001
	Inner > Outer	Recovery	3.75	3.58	3.92	<0.001
	Inner > Outer	SF	3.85	3.20	4.64	<0.001

Marker20	Comparison	Region	log(FC/OR)	2.5% CI	97.5% CI	p-value
HIF	HF > Recovery	Inner	-0.13	-1.21	1.04	0.776
	HF > SF	Inner	0.31	-0.89	1.50	0.537
	Recovery > SF	Inner	0.44	-0.83	1.61	0.409
	HF > Recovery	Outer	0.45	-0.65	1.61	0.387
	HF > SF	Outer	1.02	-0.20	2.22	0.083
	Recovery > SF	Outer	0.57	-0.71	1.73	0.275
	Inner > Outer	HF	0.25	0.11	0.39	<0.001
	Inner > Outer	Recovery	0.82	0.72	0.92	<0.001
	Inner > Outer	SF	0.95	0.81	1.09	<0.001
Zinc	HF > Recovery	Inner	-0.07	-2.45	2.41	0.94
	HF > SF	Inner	-1.20	-3.63	1.38	0.31
	Recovery > SF	Inner	-1.11	-3.64	1.54	0.37
	HF > Recovery	Outer	-1.48	-3.86	0.99	0.212
	HF > SF	Outer	-2.59	-5.02	0.00	0.05
	Recovery > SF	Outer	-1.11	-3.61	1.49	0.351
	Inner > Outer	HF	0.28	0.12	0.44	0.001
	Inner > Outer	Recovery	-1.11	-1.24	-0.99	<0.001
	Inner > Outer	SF	-1.13	-1.32	-0.93	<0.001
MFN2	HF > Recovery	Inner	-0.41	-0.84	0.08	0.108
	HF > SF	Inner	-0.26	-0.67	0.15	0.198
	Recovery > SF	Inner	0.16	-0.39	0.66	0.644
	HF > Recovery	Outer	-0.42	-0.83	0.08	0.114
	HF > SF	Outer	-0.54	-0.95	-0.12	0.018
	Recovery > SF	Outer	-0.12	-0.67	0.38	0.636
	Inner > Outer	HF	0.20	0.17	0.24	<0.001
	Inner > Outer	Recovery	0.20	0.16	0.25	<0.001
	Inner > Outer	SF	-0.08	-0.11	-0.04	<0.001
p16	HF > Recovery	Inner	2.05	-2.79	6.46	0.383
	HF > SF	Inner	-0.91	-5.70	3.73	0.716
	Recovery > SF	Inner	-3.02	-8.76	3.51	0.33
	HF > Recovery	Outer	2.58	-2.38	7.04	0.276
	HF > SF	Outer	-1.33	-6.07	3.31	0.604
	Recovery > SF	Outer	-3.97	-9.71	2.59	0.206
	Inner > Outer	HF	0.03	-0.24	0.30	0.799
	Inner > Outer	Recovery	0.56	0.21	0.90	0.001
	Inner > Outer	SF	-0.37	-0.58	-0.17	<0.001

Table S1. Relative statistical differences in region-specific biomolecular expressions: Log-fold change/odds ratios of relative differences in expression/positivity between conditions, stratified by their region of origin; * MFN2 differences are log-fold change expression whereas rest are positivity/odds ratio based

Marker	Δ-log(FC/OR)	2.5% CI	97.5% CI	p-value
**HIF**	-0.133	-0.306	0.037	0.134
**Ihh**	-0.109	-0.915	0.584	0.753
**PIEZO1**	-1.743	-2.843	-0.886	<0.001
**TRPV4**	0.291	-0.131	0.687	0.157
**Zinc**	0.015	-0.214	0.257	0.887
**p16**	0.932	0.528	1.338	<0.001
**MFN2**	0.279	0.211	0.348	<0.001

Table S2. Region-specific statistical differences in biomolecular expressions between recovery and soft food conditions: Differences (Δ) between recovery and soft food conditions in log-fold change/odds ratios of relative differences in expression/positivity between inner and outer regions; * MFN2 differences are log-fold change expression whereas rest are positivity/odds ratio-based the reference group is recovery.

Name		Targeted Channel	Treatment	
			Dose	Duration
Yoda1	Agonist	PIEZO1	5 µM	2 hrs
GsMTx4	Antagonist	PIEZO1	0.5 µM	2 hrs
GSK1016790A	Agonist	TRPV4	10 nM	2 hrs
GSK205	Antagonist	TRPV4	10 µM	2 hrs

Table S3. PIEZO1 and TRPV4 agonists and antagonists. Dosage and duration for Yoda1 (PIEZO1 agonist), GsMTx4 (PIEZO1 antagonist), GSK1016790A (TRPV4 agonist), and GSK205 (TRPV4 antagonist).

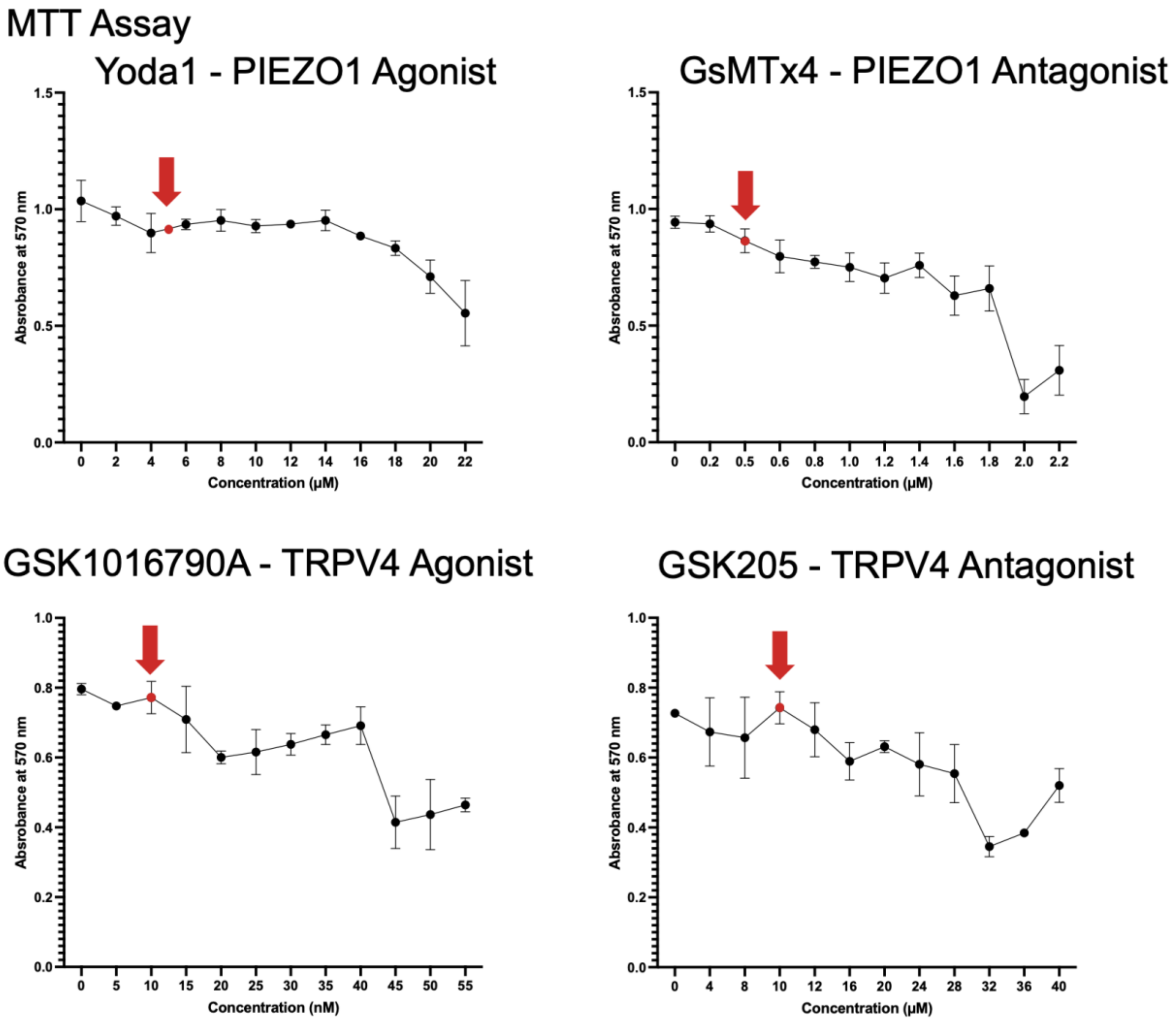

Figure S2. MTT assay. Cellular metabolism and viability were determined for various concentrations of Yoda1 (PIEZO1 agonist), GsMTx4 (PIEZO1 antagonist), GSK1016790A (TRPV4 agonist), and GSK205 (TRPV4 antagonist). Concentrations used for each treatment are indicated by the red arrow. None of the concentrations used in ATDC5 experiments had significant effects on MTT reduction.

Primer	Sequence (5'-3')	Size
L-19	Forward: GAGTCCCGGAAGACGAGAG<br>Reverse: ATCGGAAGCACCTTTCTCCT	
MFN2	Forward: AGAACTGGACCCGGTTACCA<br>Reverse: CACTTCGCTGATACCCCTGA	82
DRP-1	Forward: CAGGAATTGTTACGGTTCCCTAA<br>Reverse: CCTGAATTAACTTGTCCCGTGA	253
HIF-1α	Forward: GTCCCAGCTACGAAGTTACAGC<br>Reverse: CAGTGCAGGATACACAAGGTTT	136
SOD2	Forward: CAGACCTGCCTTACGACTATGG<br>Reverse: CTCGGTGGCGTTGAGATTGTT	113
PIEZO1	Forward: CTTACACGGTTGCTGGTTGG<br>Reverse: CACTTGATGAGGGCGGAAT	
TRPV4	Forward: ATGGCAGATCCTGGTGATGG<br>Reverse: GGAACTTCATACGCAGGTTTGG	214
ZIP8	Forward: GAACAATTGCCTGGATGATCACGC<br>Reverse: AAGCCGGTTAACATCCCTGCATTC	430
ZnT1	Forward: TGATCGTGGTCGTGAATGCCTTG<br>Reverse: CGAATTCAGGCTGGATGGTGGTAG	486
MT1	Forward: CGGGATGAGTTGCCGGTTC<br>Reverse: CGGGAAGTAACGACGATAACAC	208

Primer sequences:
PIEZO1: Wang et al 2020, Nat. Commun
ZIP8, ZnT1: Kim et al 2014, Cell
All other primer sequences are from PrimerBank, Harvard Medical School

Table S4. Real-time quantitative polymerase chain reaction primers

Agonist/Antagonist	Marker	Δ-log(FC)	2.5%CI	97.5% CI	p-value
PIEZO1	PIEZO1	-0.52	-0.89	-0.11	0.02
	TRPV4	-0.19	-0.68	0.33	0.407
	MFN2	0.85	0.44	1.26	0.001
	DRP-1	1.72	1.27	2.13	<0.001
	ZIP8	2.40	1.97	2.81	<0.001
	MTF-1	3.03	2.68	3.36	<0.001
	HIF1	-0.34	-0.63	-0.03	0.0395
	SOD2	-0.73	-0.99	-0.48	<0.001
	ZnT1	-0.83	-1.23	-0.44	0.002
	ZnT5	-1.40	-1.74	-1.08	<0.001
TRPV4	PIEZO1	-0.86	-0.98	-0.73	<0.001
	TRPV4	-0.84	-1.20	-0.46	0.001
	MFN2	-1.85	-2.16	-1.54	<0.001
	DRP-1	-2.19	-2.42	-1.93	<0.001
	ZIP8	-3.18	-3.40	-2.97	<0.001
	MTF-1	-2.95	-3.11	-2.79	<0.001
	HIF1	-0.08	-0.32	0.15	0.454
	SOD2	0.40	-0.02	0.79	0.064
	ZnT1	0.42	0.01	0.82	0.047
	ZnT5	-1.27	-0.19	-2.19	0.03

Table S5. Differences (Δ) between Day 7 minus Day 5 in log-fold change representing relative differences in marker expression between agonist minus antagonist conditions

1a. *In vitro* – D5 Agonist

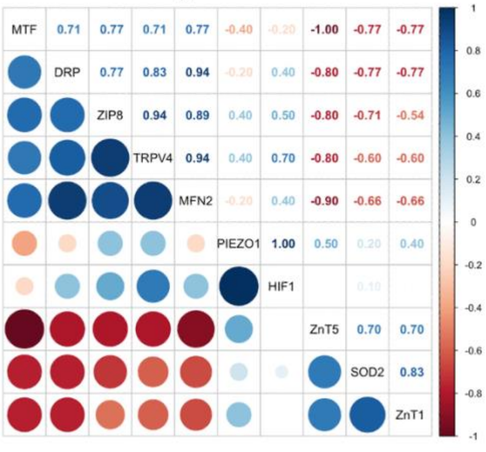

1a. *In vitro* – D7 Agonist

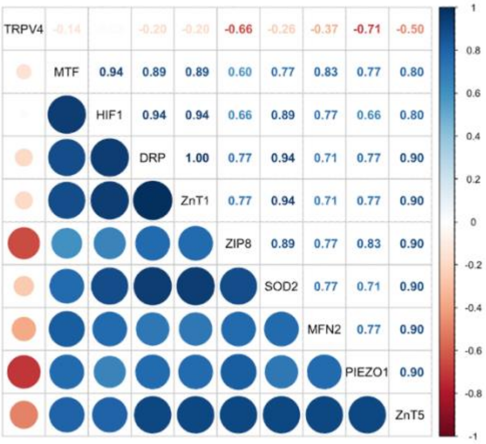

1b. *In vitro* – D5 Antagonist

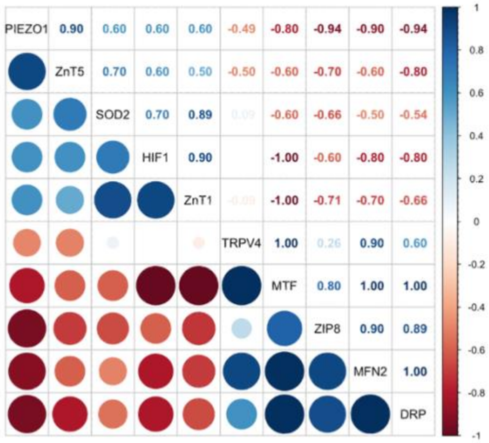

1b. *In vitro* – D7 Antagonist

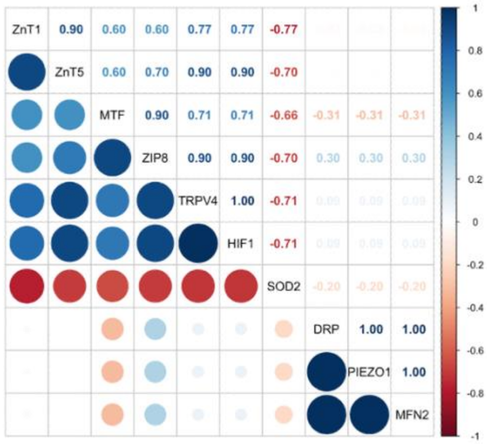

Figure S3. Spearman correlation for *in vitro* data

**Table S6.** Relative differences with vehicle in Log(FC) at days 5 and 7							
Ag_Ant_Marker	Marker	day	ag_ant	Fold Change	2.5% CI	97.5% CI	p-value
**PIEZO1**	PIEZO1	5	Ag	1.82	1.46	2.27	0.000
**PIEZO1**	PIEZO1	5	Antg	0.84	0.69	1.02	0.083
**PIEZO1**	PIEZO1	7	Ag	1.47	1.22	1.79	0.001
**PIEZO1**	PIEZO1	7	Antg	1.14	0.94	1.40	0.168
**TRPV4**	PIEZO1	5	Ag	5.33	4.82	5.85	0.000
**TRPV4**	PIEZO1	5	Antg	3.30	3.03	3.60	0.000
**TRPV4**	PIEZO1	7	Ag	0.34	0.32	0.37	0.000
**TRPV4**	PIEZO1	7	Antg	0.50	0.46	0.54	0.000
**PIEZO1**	TRPV4	5	Ag	2.29	1.69	3.09	0.000
**PIEZO1**	TRPV4	5	Antg	1.52	1.10	2.10	0.015
**PIEZO1**	TRPV4	7	Ag	0.96	0.73	1.28	0.774
**PIEZO1**	TRPV4	7	Antg	0.77	0.57	1.03	0.071
**TRPV4**	TRPV4	5	Ag	7.86	6.21	9.94	0.000
**TRPV4**	TRPV4	5	Antg	1.97	1.54	2.51	0.000
**TRPV4**	TRPV4	7	Ag	0.61	0.48	0.78	0.000
**TRPV4**	TRPV4	7	Antg	0.35	0.28	0.45	0.000
**PIEZO1**	MFN2	5	Ag	1.12	0.87	1.46	0.331
**PIEZO1**	MFN2	5	Antg	2.53	1.91	3.32	0.000
**PIEZO1**	MFN2	7	Ag	1.39	1.09	1.77	0.010
**PIEZO1**	MFN2	7	Antg	1.36	1.07	1.74	0.017
**TRPV4**	MFN2	5	Ag	9.70	8.02	11.53	0.000
**TRPV4**	MFN2	5	Antg	2.49	2.08	2.98	0.000
**TRPV4**	MFN2	7	Ag	0.27	0.22	0.33	0.000
**TRPV4**	MFN2	7	Antg	0.45	0.37	0.53	0.000
**PIEZO1**	DRP-1	5	Ag	0.85	0.64	1.11	0.215
**PIEZO1**	DRP-1	5	Antg	3.04	2.31	4.02	0.000
**PIEZO1**	DRP-1	7	Ag	2.24	1.71	2.92	0.000
**PIEZO1**	DRP-1	7	Antg	1.43	1.09	1.92	0.013
**TRPV4**	DRP-1	5	Ag	11.54	9.23	14.47	0.000

**TRPV4**	DRP-1	5	Antg	1.81	1.45	2.27	0.002
**TRPV4**	DRP-1	7	Ag	0.35	0.28	0.44	<0.001
**TRPV4**	DRP-1	7	Antg	0.49	0.39	0.61	<0.001
**PIEZO1**	ZIP8	5	Ag	0.50	0.38	0.65	<0.001
**PIEZO1**	ZIP8	5	Antg	2.83	2.13	3.73	<0.001
**PIEZO1**	ZIP8	7	Ag	3.38	2.63	4.33	<0.001
**PIEZO1**	ZIP8	7	Antg	1.76	1.31	2.34	0.001
**TRPV4**	ZIP8	5	Ag	8.47	7.37	9.65	<0.001
**TRPV4**	ZIP8	5	Antg	0.81	0.70	0.92	0.006
**TRPV4**	ZIP8	7	Ag	0.15	0.13	0.18	<0.001
**TRPV4**	ZIP8	7	Antg	0.35	0.31	0.40	<0.001
**PIEZO1**	MTF-1	5	Ag	0.22	0.18	0.26	<0.001
**PIEZO1**	MTF-1	5	Antg	2.20	1.78	2.71	<0.001
**PIEZO1**	MTF-1	7	Ag	7.61	6.29	9.13	<0.001
**PIEZO1**	MTF-1	7	Antg	3.75	3.12	4.51	<0.001
**TRPV4**	MTF-1	5	Ag	10.60	8.31	13.09	<0.001
**TRPV4**	MTF-1	5	Antg	1.04	0.81	1.30	0.732
**TRPV4**	MTF-1	7	Ag	0.14	0.11	0.16	<0.001
**TRPV4**	MTF-1	7	Antg	0.25	0.20	0.30	<0.001
**PIEZO1**	HIF1	5	Ag	0.93	0.73	1.19	0.499
**PIEZO1**	HIF1	5	Antg	0.66	0.51	0.84	0.004
**PIEZO1**	HIF1	7	Ag	1.44	1.17	1.76	0.002
**PIEZO1**	HIF1	7	Antg	1.45	1.19	1.77	0.002
**TRPV4**	HIF1	5	Ag	1.56	1.31	1.83	<0.001
**TRPV4**	HIF1	5	Antg	2.20	1.87	2.61	<0.001
**TRPV4**	HIF1	7	Ag	0.48	0.41	0.57	<0.001
**TRPV4**	HIF1	7	Antg	0.73	0.63	0.87	0.003
**PIEZO1**	SOD2	5	Ag	1.25	1.05	1.48	0.016
**PIEZO1**	SOD2	5	Antg	0.48	0.40	0.57	<0.001
**PIEZO1**	SOD2	7	Ag	0.95	0.79	1.13	0.500
**PIEZO1**	SOD2	7	Antg	0.76	0.64	0.91	0.006

**TRPV4**	SOD2	5	Ag	1.28	0.98	1.65	0.067
**TRPV4**	SOD2	5	Antg	2.63	2.01	3.35	<0.001
**TRPV4**	SOD2	7	Ag	0.52	0.40	0.65	<0.001
**TRPV4**	SOD2	7	Antg	0.71	0.55	0.92	0.010
**PIEZO1**	ZnT1	5	Ag	1.61	1.11	2.31	0.018
**PIEZO1**	ZnT1	5	Antg	0.66	0.45	0.97	0.031
**PIEZO1**	ZnT1	7	Ag	1.92	1.27	2.96	0.004
**PIEZO1**	ZnT1	7	Antg	1.81	1.20	2.72	0.011
**TRPV4**	ZnT1	5	Ag	0.43	0.30	0.65	0.001
**TRPV4**	ZnT1	5	Antg	1.53	1.03	2.28	0.035
**TRPV4**	ZnT1	7	Ag	0.22	0.15	0.33	<0.001
**TRPV4**	ZnT1	7	Antg	0.53	0.35	0.81	0.006
**PIEZO1**	ZnT5	5	Ag	1.14	0.90	1.44	0.215
**PIEZO1**	ZnT5	5	Antg	0.24	0.19	0.31	<0.001
**PIEZO1**	ZnT5	7	Ag	0.85	0.65	1.09	0.190
**PIEZO1**	ZnT5	7	Antg	0.73	0.55	0.95	0.028
**TRPV4**	ZnT5	5	Ag	0.55	0.36	0.87	0.016
**TRPV4**	ZnT5	5	Antg	2.01	1.29	3.11	0.008
**TRPV4**	ZnT5	7	Ag	0.33	0.21	0.51	<0.001
**TRPV4**	ZnT5	7	Antg	0.34	0.22	0.51	<0.001

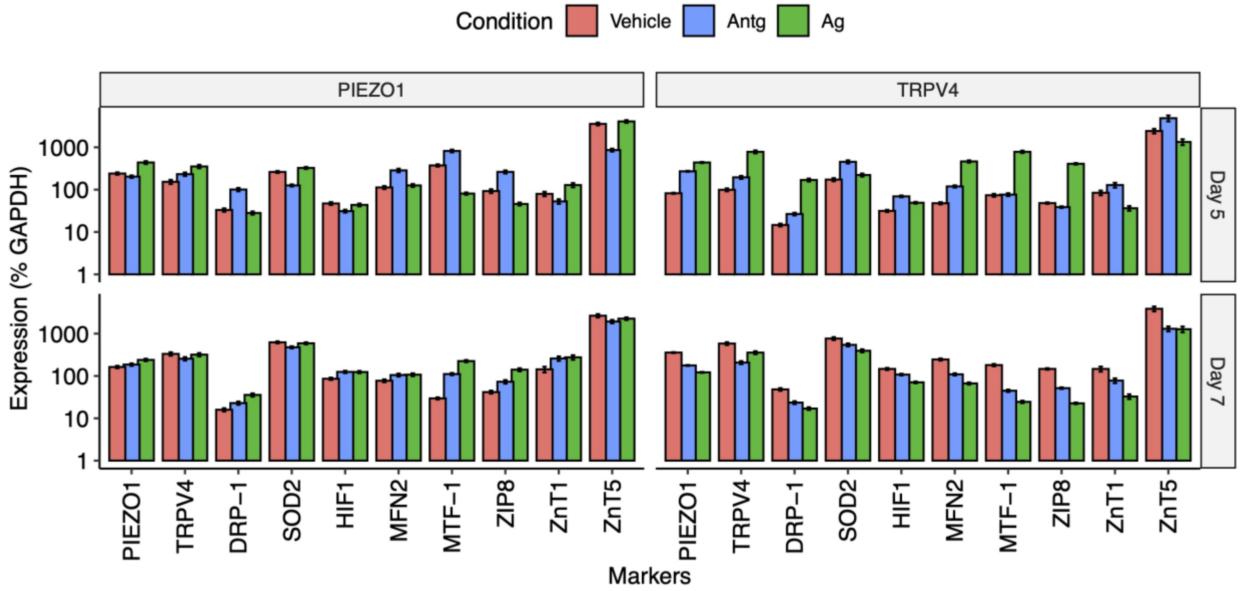

Figure S4. The relative expressions of PIEZO1 and TRPV4 (mechanosensory ion channels), SOD2, DRP-1, MTF-1, HIF-1α, and MFN2 (metabolism), ZnT1, ZnT5, and ZIP8 (zinc transporters) from ATDC5 cells from vehicle (red), agonist (green), and antagonist (blue) groups are shown in log scale.

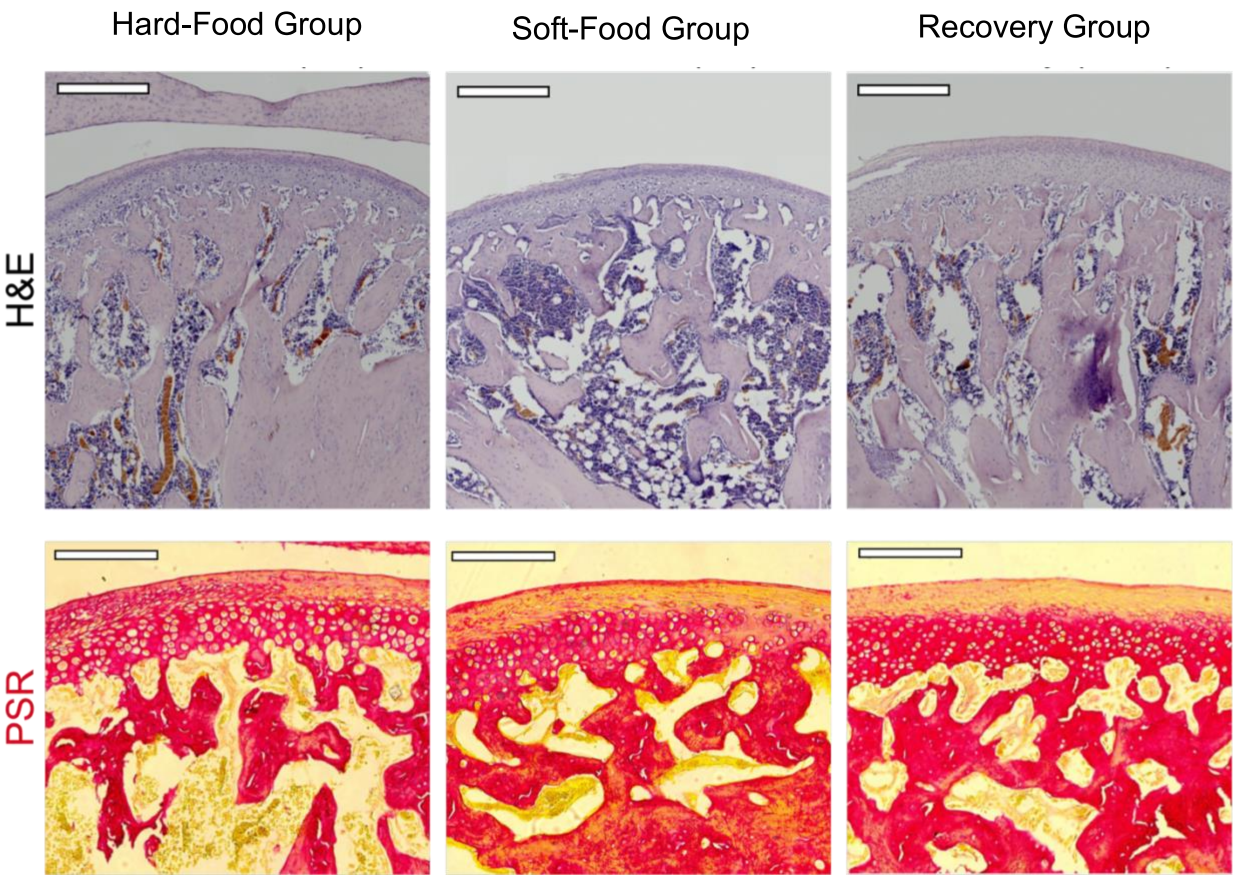

Figure S5. Outer and Inner Condylar Regions: Top and bottom rows illustrate distinct zone-specific cell distributions within the inner and outer layers. Inner layer is identified by hypertrophic and subchondral cells, while outer layer is identified by cells within the proliferative and fibrous zones. PSR stain illustrates changes in collagen in SF and REC compared to HF group. H&E Scale bar = 400μm. PSR Scale bar = 200μm.
